# Neonatal care in rural Karnataka: healthy and harmful practices, the potential for change

**DOI:** 10.1186/1471-2393-9-20

**Published:** 2009-05-20

**Authors:** Amy J Kesterton, John Cleland

**Affiliations:** 1 Centre for Population Studies, London School of Hygiene and Tropical Medicine, 50 Bedford Square, London, WC1B 3DP, UK

## Abstract

**Background:**

Every year four million babies die in the first month of life and a quarter of these take place in India. A package of essential newborn care practices exists, which has a proven impact on reducing mortality, and can be implemented in low resource settings. However, childbirth and the neonatal period are culturally important times, during which there is strong adherence to traditional practices. Successful implementation of the package therefore requires in-depth knowledge of the local context and tailored behaviour change communication.

**Methods:**

This study was carried out in rural Karnataka, India. It uses quantitative data from a prospective survey following mothers through their experience of pregnancy and the postnatal period; and qualitative data from in depth interviews and focus group discussions conducted with mothers, grandmothers and birth attendants. It explores local newborn care practices and beliefs, analyses their harmful or beneficial characteristics and elucidates areas of potential resistance to behaviour change and implementation of the essential newborn care package.

**Results:**

Findings show that many potentially harmful newborn care practices are being carried out in the study area, such as unhygienic cord cutting, delayed breastfeeding and early bathing. Some are more amenable to change than others, depending on the strength of the underlying beliefs, and acceptability of alternative care. However, movement away from traditional practices is already taking place, particularly amongst the more educated and better off, and there is a clear opportunity to broaden, direct and accelerate this process.

**Conclusion:**

Community education should be a focus of the National Rural Health Mission (NRHM) and Integrated Management of Neonatal and Childhood Illness (IMNCI) program being implemented in Karnataka. The added capacity of the new Accredited Social Health Activists (ASHAs) could enable more women to be reached. With careful tailoring of behaviour change messages to the local context, government outreach workers can become effective brokers of positive change and significant improvements in home newborn care and neonatal mortality are possible.

## Background

### Introduction

Globally four million deaths occur every year in the first month of life [1,2]. While under-5 mortality has declined in recent decades, the bulk of this improvement has been at later ages and a lack of progress in the neonatal period is causing stagnation. Neonatal deaths now represent 40% of those to under–fives. To reach Millennium Development Goal 4 to reduce child mortality to two thirds of its 1990 level by 2015, this problem needs urgent attention. With a quarter of neonatal deaths taking place in India, this country is integral to any successful global effort [1,3].

Infectious diseases are associated with 30–40% of neonatal deaths. Other major direct causes are preterm delivery and birth asphyxia, with low birth weight and hypothermia representing important contributing factors in many deaths [4-6]. Care practices at and immediately following delivery can contribute to newborn morbidity and mortality, but a package of essential newborn care (ENC) practices has been proven to reduce these risks [7,8]. These practices include cleanliness in delivery; the use of a clean, sterilised blade to cut the cord, and tying with clean string; immediate drying and wrapping of the newborn and delaying of first bath; initiation of exclusive breastfeeding within an hour and the prompt resuscitation of asphyxiated newborns [7,9]. It is estimated that 70% of deaths could be avoided by universal application of this ENC package, which can be implemented in low resource settings without easy access to skilled medical attendance [10]. With the current level of institutional delivery in India at 40.7% [11] these features are highly important.

The ENC interventions need to be tailored to the local environment. The time around childbirth is culturally very important and existing behaviours are commonly rooted in traditional beliefs. An understanding of newborn care practices is therefore essential if effective behaviour change strategies are to be developed [6,12,13]. Within India the wide religious, cultural, regional and socio-economic variations make adaptation of the package particularly important. Improvement of care practices through the use of village health workers [14] and women's groups [15] has already been demonstrated to reduce mortality in particular South Asian settings.

### Aims and objectives

The aim of this paper is to explore local newborn care practices in the study area of rural Karnataka, India, identify those that may be harmful and beneficial and assess their mutability. This involves specifying the beliefs, motivations and situational constraints which maintain particular behaviour patterns; and a consideration of the potential for change and acceptance of the essential newborn care interventions.

### Government policy

The Indian government included the training of physicians and Traditional Birth Attendants (TBAs) in essential newborn care as part of their Child Survival and Safe Motherhood Programme (CSSM) launched in 1992. In 1997 this gave way to the Reproductive and Child Health Programme (RCH) and ENC training was extended to the Auxiliary Nurse Midwives (ANMs), who are the most peripheral salaried service providers providing maternal and child health care services, including delivery care [3].

Since the research for this paper was conducted, further policy developments have taken place. The second five-year phase of the RCH programme (2005-10) is now being implemented which contains a comprehensive newborn strategy. This includes implementation of the Integrated Management of Neonatal and Childhood Illness (IMNCI). In this government outreach workers, the ANMs and Anganwadis (child health and nutrition workers, whose responsibilities include breastfeeding counselling) are mandated to visit all neonates at home three times within the first ten days to provide home based preventive care, health promotion and early detection of sickness requiring referral [16,17]. This outreach model is based on work using village health workers in Maharashtra by the organisation SEARCH [14]. IMNCI was piloted in six districts in 2003–2004 and has now been taken up by several state governments, including Karnataka [3,17].

Closely linked to IMNCI is the National Rural Health Mission, also initiated in 2005, which states the reduction of infant mortality as one of its key objectives. The Mission includes the Accredited Social Health Activist Programme (ASHA), which aims to train one activist per village to generate awareness of health and its social determinants and to mobilise the community towards taking action. It is a national program, but focuses on 18 states deemed to have weak health systems and poor socio-demographic status. Although Karnataka is not a focus state, neonatal mortality is rather high despite progress in other demographic and socioeconomic indicators, and efforts are being made to implement the NRHM. Anganwadi workers and ANMs already operate at the village level and the intention is for ASHAs to work under the guidance of the Angandwadi, but reporting ultimately to the ANM. Some conflict between these workers has been reported, and there is real need for clear roles and responsibilities to be enforced. If organised carefully the potential for the increased impact of collaborative working is great [18].

## Methods

The research for this paper was carried out with the Belaku Trust, a South Indian Community Health NGO based in Bangalore rural district, Karnataka. The study area consists of 11 villages with a population of about 6000 households surrounding Kanakapura town, 60 kms from Bangalore. This study encompasses complementary quantitative and qualitative data.

### Quantitative data

Secondary analysis of an Obstetric Survey conducted between 1996 and 1998 by the Belaku Trust has been carried out. The survey explored obstetric morbidity, health seeking behaviour, factors affecting service uptake and traditional beliefs and practices in the childbearing period. All women in the study area who were already pregnant at the start of the study, or who became pregnant during the study period, were voluntarily enrolled in the survey until the required total of 500 women was reached. Trained field workers fluent in Kannada, the local language, administered a series of 5 pre-tested questionnaires (three antenatally, one three to five days post delivery and one later in the post-natal period). A prospective research design was implemented in order to avoid the recall biases inherent in retrospective studies. Only 388 were re-interviewed immediately after delivery, with loss universally due to women returning to their natal village for delivery. No significant differences were found between the drop out and retained groups and thus any bias from sample attrition is likely to be minor. Previous analysis has concentrated on the mother [19-21], but this paper utilises the data relating to the newborn. This allowed the prevalence of relevant care practices to be documented and gave an overview to guide the more in-depth qualitative work.

### Qualitative data

An anthropological approach was deemed most appropriate for a more in-depth exploration of existing newborn care practices, to gain understanding of the norms and beliefs that drive them, and assess their mutability. The first author lived in the field site between August and March 2004–5 while the primary qualitative fieldwork was carried out. Semi-structured In-Depth Interviews (IDIs) and Focus Group Discussions (FGDs) were conducted by a trained Kannada-English speaking fieldworker using pre-tested guides. Both followed a similar format, background information on education, socio-economic and reproductive health status was gathered followed by questions to elucidate the practices associated with the birth and care of participant's last child. These were explored to try and glean the beliefs and reasoning behind behaviours and potential barriers to change. Finally, reactions to recommended ENC practices were investigated, to help determine their acceptability and ascertain barriers to change. The use of IDIs and FGDs allowed in-depth, one to one, more private communication, and lively group dynamics and discussion to be benefitted from in data collection. To minimise courtesy bias every effort was made to make people feel at ease and express their own opinions freely in a two-way transfer of ideas. In total eight FGDs were carried out, three with groups of mothers, two with elders, one with birth attendants and two with mixed age groups. Thirty nine IDIs were conducted, 13 with recently delivered mothers, ten with grandmothers, nine with birth attendants and seven with key informants chosen for their particular experience or insight.

People from a range of educational, caste and social groups were chosen purposively for their heterogeneity and selection was informed by the emerging data. Data collection was continued until saturation was reached, defined as 'data adequacy', the point at which no new information is being obtained [22]. The only possible hazard of participation was psychological stress caused by talking about newborn mortality. To avoid this, participation was voluntary and respondents could decline to talk about particular topics. However, nobody approached chose not to participate or to not talk about all topics. Discussing and obtaining written informed consent was given plenty of time before the interview and ethical clearance was obtained from the London School of Hygiene and Tropical Medicine.

IDIs and FGDs were all audio-taped and full transcripts translated into English. Analysis began during data collection, when transcripts and field notes were read and emerging themes noted and monitored. This allowed adjustments to be made along the way and ideas to be tested. This cyclical process is taken from the grounded theory approach. However, framework analysis was used as the main method of analysing the data as it is explicitly geared towards generating findings relevant to policy and practice, and is popular with health and social researchers for this reason [23]. The integrity of individual respondents account's is preserved throughout the analysis to maintain the authenticity of participants' narratives while eliciting the broader meaning and relating it to the objectives. Familiarisation with the data is the first step; thematic analysis was then carried out to develop a coding scheme, the codes were then applied to the whole dataset; and a summary of the data was arranged across cases and under themes. The final stage of mapping and looking at the relationships between codes is what moves framework analysis beyond a sophisticated thematic analysis [22]. Awareness and exploration of the ways in which the researcher's involvement in the study influences, and informs construction of meanings throughout, has helped take account of the role of the researcher. In collection, analysis and interpretation this was also combated by seeking the opinion and viewpoint of the interviewer, who was from the local area and very familiar with the field site. Participants' confidentiality and anonymity was maintained throughout. Quotations which most clearly express the broad findings in different themes are largely presented in this article. When relevant these are combined with the analysis of additional quotes which represent 'deviant' cases or those that are outside the norm.

## Results

### Characteristics of the study area

**Figure 1:**
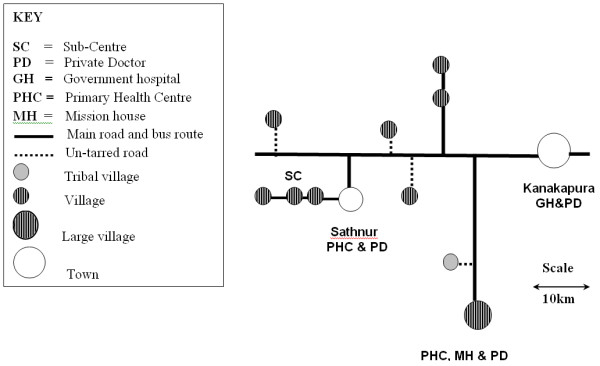
**Map to show location of the 11 study villages and local health care services**.

The locations of the 11 villages in relation to health care services are shown in Figure 1, women from all of these were involved in both the Obstetric Survey and later qualitative work.

As the sub-centres are not consistently staffed access to primary health centres (PHCs) or hospitals is important. Three of the villages are close to Sathnur PHC, the large village has its own PHC and the others are in a position to either access Kanakapura hospital or to go to Sathnur. Only in the three villages close to Sathnur is regular professional antenatal, delivery and postnatal home care available, provided by a single diligent ANM. Traditional birth attendants (TBAs) or dais are present in all except the tribal village; they tend to be old and consequently lowly educated, and generally provide their services for free, or a small sum. Some have received training. There are also many women who have experience of delivery but do not formally consider themselves TBAs. It is actually much more common for an experienced relative or neighbour to conduct delivery. Water is accessed from common water tanks, filled from a bore well in each village.

The Obstetric survey data (see Table 1) gives an overview of the characteristics of the women living in these villages. Forty one percent of mothers were in their teens and for 42% it was their first child. Education levels are low with almost 45% of women having received no formal education.

**Table 1:** Background characteristics of mothers in the rural Karnataka study area

**Background**	**Percentage of study sample**	
**Age of mother**	**1st births **	**All births**

14–19	46.3	41.3
20–24	42.2	44.2
25+	11.6	14.5
Total (n)	100(147)	(387)

**Birth Order**		

1st	42.1	
2nd	32.7	
3rd	14.8	
4^th ^or higher	10.4	
Total (n)	100 (385)	

**Caste**		

Scheduled tribe (Lambani)	5.0	
Scheduled caste	26.1	
Other backward caste	68.9	
Total (n)	100 (383)	

**Mother's Education**		

No education	44.6	
1–5 (primary)	12.6	
6–8 (secondary)	18.8	
9+ (tertiary)	24.0	
Total (n)	100 (383)	

**Wealth **Household possessions (Rupees 1 US$=~ 47 Rupees)		

<1000	46.9	
1001–5000	34.0	
5001–15000	11.3	
>15000	7.7	
Total (n)	100 (388)	

The village inhabitants are all from broadly low caste groupings. Except for the tribal village all villages have a mixture of Other backward Caste (Obc) and Scheduled Caste (SC) members. Obc is the most populous group encompassing landowning agricultural castes that are typically the most socio-economically dominant people in the villages. There is also a significant minority of SC members, which form the lowest group in the caste hierarchy and in socio-economic status. They live in a separate colony within the villages. Finally there are the Scheduled Tribe (ST) members. The Lambani, are the only tribal group in the area, they are confined to their own village and widely regarded as the most disadvantaged. They were originally a nomadic group and have distinct tribal characteristics in their dress, language and customs, including in relation to health seeking and morbidity.

Caste separatism (refusal to intermarry, eat with one another, or touch one another) is strongly followed in rural India. Beliefs about ritual purity and impurity are all-pervasive and important rationale for this separatism. Generally, high status is associated with purity and low status with pollution. Some actions are thought to be too defiling for certain castes to perform, and some castes are thought to be too impure to perform certain other activities [24]. Scheduled caste members are seen as below the level of pollution, and as a servicing caste. The touching of a scheduled caste member traditionally amounts to pollution, they are not allowed to enter the Hindu temple and others will not accept food or water from them. Involvement with the process of childbirth is seen as ritually polluting, an important theme in determining behaviour in this period [25,26].

### Neonatal care practices

#### Banthana

The post-partum period or 'banthana' is culturally well recognised and lasts from delivery for several months. The one-month neonatal period is not an important benchmark. Delivery, as in much of rural India, is considered an impure or ritually 'polluting process' from which cleansing is needed. Both mother and child are in a state of pollution and vulnerability beginning at birth or exposure to blood or birth related fluids from which cleansing and recuperation is needed [12,27]. The mother and child are thought to be physically and psychologically vulnerable to illnesses of natural and supernatural origin. They may be affected by 'drishti' (evil eye), or possessed by spirits and afflicted with illnesses such as '*bheeti shanke*' (literally meaning terror and superstition). Banthana is divided into discrete periods marked by changes in rituals, diet, mobility, seclusion and health promotive and preventive measures aimed at protecting the mother's and baby's health. Tradition, ritual pollution, and lack of experience mean mothers have relatively little autonomy over newborn care and grandmothers and elders are important decision makers and care takers. Table 2 shows the traditional banthana practices relevant to the newborn.

**Table 2:** Banthana practices relevant to the newborn

**Time**	**Practices**
Delivery to day 3	-Home delivery takes place in a warm, dark room and mother and child are then confined to it
	-Umbilical cord cut after placenta is delivered
	-Mother and child are kept warm, although child may be left exposed until placenta is delivered
	-Access to mother and child is limited to care givers and immediate family
	-Pre-lacteal feeds are given to the baby including castor oil, sugar water and diluted cow's milk, also wet nursing
	-Sponge bath or body bath given for both infant and mother

Day 3 to 40	-Traditionally breastfeeding is initiated after the mother has a head bath on the third day (it is a common belief that milk 'descends' after the head bath). Head baths are given regularly to mother and child until Day 40
	-Ritual pollution period ends on Day 40 (40 days is the important length, the 28 day neonatal period has no meaning)

Day 40 to 3/4 months	-After visiting the temple mother and child can leave the house for essential visits

#### Place of Delivery

Home births are common in rural Karnataka and according to NFHS-2 in the period 1996–8, 61.4% of births took place outside of a health facility [28]. In the Obstetric Survey home births were found to account for a representative 65%, and although the qualitative data suggests this is reducing, preference for delivery in the familiar home environment, is still common. It is traditional for a mother to return to her natal home, particularly for a first delivery. In institutional deliveries, hospital stays are short, so most newborn care still takes place at home. There is significant room for improvement in quality of care. Mothers who cannot pay in full (or generously) are often treated roughly and in general little or no advice is given regarding home care of the baby, even when the child is under weight or delivery complicated.

Table 3 shows attendance at institutional and home deliveries. The home deliveries with an ANM or nurse are heavily concentrated in the three villages nearest to Sathnur PHC, served by the very active ANM. She lives in one of the villages and is therefore easily accessible. In the other villages, it is more common to have a relative/neighbour or TBA.

**Table 3:** Assistance at delivery from the Obstetric Survey

	Institutional delivery (%)	Home delivery (%)
Government doctor	19.7	

Private doctor	17.5	

ANM/Nurse	62.8	37.0

TBA		12.4

Relative/neighbour		48.2

No attendant		2.4

Total	100 (n = 137)	100 (n = 251)

In the study almost half of home deliveries are conducted by a relative or neighbour, with many women having experience of deliveries but not considering themselves TBAs. In the tribal village it is exclusively the case. Work in other areas of India has shown that the term 'dai' or TBA is rarely used locally as there is no such clearly defined group, and it stems partly from use by the government in their training programs [29]. In the study area there are scheduled caste women who consider themselves TBAs and conduct deliveries and postnatal care. However, in some communities of Northern India, TBAs often do not get involved until after delivery, which is usually carried out by a family member. TBAs are at least certainly not in full charge. The TBAs are scheduled caste and thought of as inferior, they carry out the more menial jobs such as cutting the cord, disposing of the placenta and cleaning soiled items, due to the associated pollution [30]. In other places though, including the study area, their role is not only associated with 'pollution' and they also help advise on postnatal care in the period of vulnerability and recuperation for mother and child [31]. TBAs can be both stigmatized and revered for their work [29]. In the study area TBAs were found to have a real sense of pride in their work and defend it protectively; they are particularly strongly attached to traditional beliefs and practices, which often conflict with those recommended by the ENC package [8]. Many who have received training still express a preference for the comfort and simplicity of their old methods.

#### Delivery hygiene

Traditionally cleanliness in home delivery has not been considered a priority in the study area. In the past mothers would squat on the floor, often in the cowshed. Customs have changed in most places and it is now normal to deliver in the house and to prepare an area with a mat or some sacking. However, as the quotation below shows advanced planning is typically quite limited. There is also an emphasis on ease of cleaning after delivery. This was typified by one TBA who said, she insists the mother lies on hay and sacking, which can be thrown away.

*Well I don't know when it is going to happen so I might not always keep a clean place for the delivery *(Grandmother, IDI).

In the tribal village strong beliefs about god's presence in the house prohibit delivery there. Childbirth still takes place in the cowshed, which is separate from the house, and also easier to clean afterwards.

Grandmother-*We do delivery in the cow shed, God is kept inside the house, in the hall and rooms etc. So we don't want to make the place dirty*.

Interviewer-*Could you do the delivery somewhere cleaner, in the house rather than the cowshed?*

Grandmother-*Yeah, yeah will you come and clean out all the blood? I am not going to do it in the house. God is inside the house and delivery is polluting so I can't do it in the house*. *You know if the mother has stomach pains right from the start we wouldn't want her to be in the house we say go and sit in the cowshed. People will make fun of us, 'they are so dirty they conducted the delivery inside the house' they will say *(Grandmother, Tribal village, IDI).

The local understanding of cleanliness is quite different from that of biomedical science [32]; it is spiritual as well as physical. In general hygiene is treated casually with little understanding of infection transmission. However, the younger generation are generally more aware, and as the quotation below shows there is some basic understanding amongst a minority, which should spread.

*I do the delivery in the room or in the hall. I'll never allow them to do it in the cowshed as it isn't clean, I tell them no I don't want to do it there we'll do it in the room itself...*. *When I learnt from my mother I learnt about being clean, I know if you're not clean they may get some pain or infection, I insist on keeping warm water and soap there and I only put my hands inside if I have to *(Untrained TBA, IDI).

#### Cord cutting

Cord cutting is of particular interest generally being indicative of broader obstetric care levels [23]. The primary birth attendant usually carries out cord cutting but a spectrum of implements is used. The Obstetric survey data in Table 4 shows that a sickle was used in almost a third of deliveries by unskilled attendants, and qualitative data suggests this is still common in the tribal village and in poor and uneducated families. In other cases, a used blade or implement is washed and reused, but never sterilised. In general there is more concern about cleaning the polluted blade after it has been used.

**Table 4:** Newborn care practices from the Obstetric survey

**Newborn Care Practice**	**Percentage**	
**Cord cutting behaviour**	**Unskilled birth attendant TBA, relative or neighbour**	**Skilled birth attendant doctor, nurse or ANM**

Attendant's own implement -Not known if sterilised	0.0	56.5

Attendant's own implement -Not sterilised	3.2	13.2

Attendant's own implement – Sterilised	2.3	0.8

New blade-Not sterilised/washed	34.1	7.0

New blade-Washed with water	18.9	8.1

Used blade- Not sterilised	6.3	1.2

Sickle- Not sterilised	28.2	1.2

Household implement-Not sterilised	2.4	11.2

Other	2.3	0.0

Do not know what used	2.4	0.8

Total (n = 388)	100 (n = 130)	100 (n = 258)

**Cord care**		

Left open	61.8	69.4

Turmeric powder	14.5	10.8

Covered with cloth	5.3	9.0

Covered with a cloth then powder applied	2.6	3.6

Tip burnt with castor oil lamp	5.3	3.0

Tip burnt and then powder applied	1.3	0.0

Antiseptic ointment	0.0	0.6

Do not know	9.2	3.6

Total (n = 242 missing = 146)	100 (n = 76)	100 (n = 166)

**Pre-lacteal feeding (first feed)**		

Castor oil	51.3	55.2

Sugar water	20.3	21.6

Diluted animal milk	20.3	12.2

Another woman's milk	1.6	5.7

Other	4.1	4.5

None	2.4	0.4

Total (n = 370 missing = 18)	100 (n = 123)	100 (n = 247)

**Initiation of breastfeeding**		

Initiation within 3 hours	23.4	24.0

Initiation between 3–24 hours	26.6	28.9

Initiation between 24 hours and 3 days	47.6	44.7

Do not know	2.4	2.4

Total (n = 370 missing = 18)	100 (n = 124)	100 (n = 246)

Grandmother-*We ourselves do the cutting with a sickle *(shows a rusty blade from in the house).

Interviewer-*Is the cutting implement cleaned?*

Grandmother-*No, we wash it after the cutting *(Grandmother, Tribal village, IDI)

However, use of a sickle is no longer the norm, and cord cutting was done with a new blade in more than half of deliveries by unskilled attendants (see Table 4). This is particularly common amongst better off families. Most interviewees in the primary qualitative study at least knew a new blade should be used, and the younger active TBAs often said they insisted on this.

*I'll ask the people to get the new blade to cut the cord, sometimes they say its okay do it with the old blade, but I insist I need the new blade *(Untrained TBA, IDI).

In deliveries with local attendants, the cord is not traditionally cut until after the placenta is delivered. There is a strong belief, especially in the tribal village, that if the cord is cut first the placenta will remain inside the mother and cause problems. This is detrimental to the newborn as it is often left exposed and unfed pending delivery of the placenta.

*The cord should never be cut before the placenta is delivered because it will cause problems for the mother. My neighbour said 'it will go up into the chest of the mother'. Until the placenta is delivered, the baby is made to sleep next to the mother on a cloth *(Grandmother, Tribal village, IDI).

Usually the placenta is expelled spontaneously, but for 5% of women in the Obstetric survey this did not happen. Local methods are used to try to prompt delivery; these include making the mother vomit, or giving her a herbal remedy known locally as *'jeera kashyam'*.

*If the placenta does not come out, we will make her swallow her hair or else the root of an old brinjal is smashed and mixed with buttermilk and given to the woman. Or, we put chillies in the fire and make her cough, if the placenta doesn't come out, then we take her to the hospital *(Elder, Tribal village, IDI).

Amongst the local attendants, there is some awareness that immediate cord cutting is recommended but great nervousness about actually doing it. In the privacy of a one to one interview a woman explained fearing being blamed if problems occurred. Attendants are under pressure to follow the local practice.

*The ANM's cut the cord immediately and when I am with them they still do this but when I am alone the villagers say if you cut it when the mother breathes in the placenta will go up into the chest and get stuck *(Untrained TBA).

Although there is little evidence of changes in practice relating to delivery of the placenta between the Obstetric Survey and qualitative research, there is a certain acceptance of hospital practices that is helping to slowly change opinion. Mothers who had experienced early cord cutting without complication were aware it is not normal in the community but less afraid of the practice.

*In the hospital as soon as the delivery was done I thought the placenta came out, but then when they came back they removed it. I thought why didn't they do it before the cord was cut? But I was too tired to think, everything was fine *(Mother, IDI).

#### Cord care

In general strict care of the cord is not rigorously adhered to, but families may apply something if it is available. In 33% of cases in the Obstetric survey some application was used (see Table 4) and qualitative findings also found only some families choosing to treat the wound. Turmeric powder has natural antiseptic properties, but it is not always stored cleanly. Burning the tip is particularly advocated by elders as it is thought to stop bleeding and prevent infection by sealing the end. However, this practice is not commonly used anymore.

*Sometimes we use turmeric powder for the wound because it dries up the wound and heals it quickly, this time we didn't though, after the bath some baby powder would get on it that's all *(Mother, IDI).

#### Asphyxia

A small proportion of babies are born asphyxiated (6% (24) of live births in the Obstetric survey), and qualitative findings show that asphyxia is a problem about which there remains a real lack of knowledge amongst mothers. Older women described various methods of dealing with it, removing phlegm from the mouth, holding the baby upside down and patting or massaging it, blowing in the ears, sprinkling and dipping it in water and making loud noises.

*I take the cow dung; put it on the chest, and then sprinkle the water and the baby will start to cry. This is the only method I use; if it will not breathe, I think it is dead *(Untrained TBA, IDI).

ANMs were found to use similar procedures, although injections are also given. Local attendants do not use mouth-to-mouth resuscitation, and while ANMs could describe the process, evidence suggests they are not all comfortable carrying it out and poor quality of care is common.

*The baby was not breathing, the ANM wrapped the baby inside my saree thinking the baby would die but the doctor scolded the ANM and asked her to try out getting it to breathe. The baby was lifted upright and patted at the back; she dipped her hand in water and patted it on the back *(Mother, IDI).

There was a mixture of reactions to recommended practices for dealing with asphyxia. Removing phlegm was already familiar to some women and patting and massaging is commonly used; however, few were familiar with mouth-to-mouth resuscitation. There was interest in the concept but some scepticism amongst older women that it could help. Some even feared it would inhibit breathing and insisted that you had to blow on the ears or head. There were also concerns about saliva transfer, due to strong traditional reservations. However, the idea of using gauze between the mouths to prevent this was acceptable.

*Yes, it is possible you can do it but don't you think your saliva will go into the baby's mouth *(Mother, IDI)

#### Thermal care (bathing/wrapping)

Concerns about the baby looking dirty, combined with the ritual pollution associated with giving birth provides strong motivation for early bathing. The quotation below demonstrates the common belief that the white layer of vernix is food that the mother has eaten incorrectly during pregnancy and must be removed.

*It should be removed, the white things are the food stuffs eaten by the mother during her delivery..... The baby should be cleaned off to make it look good *(Elder, Tribal village, IDI).

No examples of delayed bathing without the influence of an ANM were encountered and most people were sceptical of the idea. While elders feel strongly about initial cleaning due to pollution, it is mothers wanting to be clean and hygienic that is leading to more regular washing.

Elder 1-*Yes when it was born on that day we would wash off everything, head and all and wrap the baby, and then on the 11^th ^day we would wash it again*.

Elder 2-*Now every 2, 3 or 4 days the mother and child are bathed though*.

Elder 3-*Yes now they wash, wash, and clean and clean, they do everything*. (FGD)

There is a danger that recommending delayed bathing can appear contradictory to general messages about hygiene, and it is important mothers understand the vernix is not unclean and that newborns are delicate and at risk of hypothermia. Although getting the baby clean is the immediate priority after birth, some traditional appreciation of a baby's sensitive skin does exist. Mainly older women stressed that they only washed the infant gently without soap; for them the recommended practice of just wiping the baby with a warm cloth after birth does not differ significantly and was acceptable.

*Bathing does not mean I pour a lot of water on it because the baby's skin is still delicate; I just take some water and clean it. *(Untrained TBA, IDI)

The danger of 'catching cold' is also universally understood and it is normal to deliver in a dark room with no ventilation, as this is felt to be the warmest environment.

*No no no, we always keep the room dark and only a little bit of light is allowed most of the time because the child and mother may catch cold. *(Mother, IDI)

This appreciation of a newborn's vulnerability can be taken advantage of to encourage behaviour change. The diligent ANM operating in three of the villages also highlighted that there are traditionally auspicious days for bathing after birth (odd days such as the third and fifth), which she has successfully encouraged families to delay bathing until.

The baby is traditionally wrapped after bathing and placed in a bamboo tray next to the mother. It is auspicious to keep it there for three days before transferring to a cradle. Research shows it is important for mothers to hold their babies, allowing them to suckle, keep warm and bond [33]. In the privacy of an interview one ANM explained honestly that although she tries hard to give the baby to the mother the grandmother generally strongly resists adamant that the mother must rest. In the first month, the baby is picked up if it cries and for feeding but is not generally held otherwise.

ANM-*The baby is kept near the mother on the bamboo tray, although we advise they keep it with them they don't listen*.

Interviewer-*Do the mothers normally not want to hold the baby after birth?*

ANM-*The mothers are not normally that bothered but it is the grandmothers who won't allow the mother to keep the baby, if I try and give it to the mother they say, please don't ask her to carry the baby, she is so tired how can she lift the baby, please don't ask her. Some things they won't listen to they just don't understand some things. *(ANM, IDI)

#### Breastfeeding

At the time of the Obstetric survey, newborn babies were almost universally given a pre-lacteal feed, regardless of the type of delivery attendant. While there are signs that this is reducing, it remains very common. Traditionally castor oil is given (over 50% in Obstetric Survey), believed to clean out the babies insides by making them pass a stool, followed by another mother's milk.

*We believe that the baby has impure things in the stomach and so we give oil or another mother's milk for one or two days, and only after the mother is home and has been washed and the mother washed will she then feed the baby. *(Untrained TBA, IDI)

Sugar water is also now used. One ANM explained in interview that it was government protocol approximately 10 years ago and the practice diffused into the community (over 20% in Obstetric Survey). She also admitted openly that despite the change in guidelines it was still recommended by some of her colleagues. Pre-lacteal feeds are usually given on a fingertip, increasing risk of infection.

*Some time ago about 10 years, it was in the government rules, after the birth we were taught to feed the baby sugar solution, we were told in the training we weren't given a reason, but it was then followed in the hospital. More recently though we had training and they advised us not to follow it anymore, so since then it is meant to have stopped, but some of the sisters carry on the old way. It is hard for them to change suddenly. Some will say, we used to give sugar solution all the babies are fine, why do we need to change, these won't strongly recommend breastfeeding. *(ANM)

The qualitative work shows that there is now some awareness in the community that pre-lacteal feeds should be avoided and breast milk given first, but there is little urgency in initiating breastfeeding. Mother and child are polluted after birth and mothers are concerned about breastfeeding before washing, they are also tired and want to rest. There is also a strong traditional belief that there will not be any good milk straight after birth. This belief was responsible for over 60% of delayed breastfeeding in the Obstetric survey. The colostrum produced at the start, with its thick yellow consistency, is believed to be bad for the baby. At the time of the Obstetric survey almost half breastfed within 24 hours, and it is becoming increasingly common to do so before the traditional third day. The dynamic of a FGD, with many young mothers together, meant they were particularly keen to stress that practices were changing.

Focus group member-*Nowadays we don't give castor oil, it is definitely mother's milk that is given, we feel the very first needs to be taken out, but it has become the habit to give the mother's milk. Not straight away though, first she needs to come home, clean up and after sometime she can feed, within a day or the next day if the baby comes in the night. *(Mother, FGD)

However, the colostrum is still usually removed, with little awareness of its protective properties.

*It (the first milk) is a creamy yellowish colour; it should be white milk, so we threw this milk. *(Mother, IDI)

A minority of younger or better-educated women are becoming aware that the colostrum is good for the baby. The diligent ANM explained that if supported mothers will breastfeed immediately; she wipes and wraps the baby, hands it to the mother and helps her feed. Although there may be some resistance from elders, mothers are usually amenable if helped. This shows the potential for change but the problem is that this ANM is exceptional. In the hospital staff often neglect to advise on breastfeeding and leave families to their own practices, missing this vital opportunity for education. The lack of difference between feeding in deliveries with skilled and local attendants (see Table 4) demonstrates this. Once initiated, exclusive breastfeeding is the normal practice, although gripe water is sometimes given.

## Discussion

Although the gap in time between collection of the quantitative and qualitative data is a weakness, it does help gauge how practices are changing and makes triangulation possible. The survey data provides the basis for understanding some of the qualitative data and the qualitative data built on and enriched the quantitative data.

A wide variety of newborn care practices have been documented in the study area. Traditional practices have the advantage of being affordable, culturally acceptable and relevant to the local environment and socio-cultural needs, and can be used by most of the population. However, while the 'banthana' period is focussed on returning the mother to good health and looking after the baby, risky traditional practices that may have a potentially harmful effect on the newborn have been found to be common.

The ritual pollution associated with birth leads to an emphasis on minimising the spread of pollution rather than a focus on hygiene, with the biomedical transmission of infection poorly understood. This influences cord cutting, although a promising diffusion of the practice of using a new blade is now taking place. Delayed cord cutting is very common in home deliveries due to fears of obstructing placenta delivery, and resistance to change is strong. A compromise, in which the importance of keeping the baby warm while waiting for the placenta to be delivered is emphasised, may be appropriate. Asphyxia is poorly understood and managed both at home and in institutions due to lack of familiarity rather than active resistance. There is therefore great potential for improvement. There is some appreciation of the delicate nature of newborn babies but it conflicts with the desire to wash the baby after delivery to remove 'polluting' blood and make it look clean. There is already evidence of the potential for compromise in this area of care however, and once educated, families are willing to gently wipe the baby without giving a full bath.

Pre-lacteal feeds are frequently given, and breastfeeding delayed, even in institutional deliveries where support is limited. However, positive change is already taking place with breastfeeding often no longer delayed until the traditional third day, and colostrum not universally removed. There is evidence that this could be accelerated by increasing awareness of the benefits of immediate exclusive breastfeeding and therefore instilling a sense of urgency.

Research in other areas of India and South Asia, including Pakistan and Nepal, has demonstrated similar findings; and practices such as pre-lacteal feeding and immediate bathing are common to many cultures. The association of childbirth with ritual pollution, vulnerability to evil spirits and consequently seclusion and restriction of movement is also widespread [34-37]. While some similarities exist over wide regional areas, there are also many local cultural variations, as demonstrated by differences found between the tribal village and other villages in this study.

Some movement away from traditional practices is already taking place in the study area, and effort is needed to ensure existing harmful practices are not replaced with equally damaging modern ones. While it is the younger, more educated and better off, who were most positive in their reaction to the essential newborn care interventions, previous exposure to the ideas also helped. The general potential for accelerated directed behaviour change through community based education is therefore clear. An in-depth understanding of existing local care practices and the beliefs that underlie them, as generated in this study, is crucial in informing health promotion. While mothers are often more amenable to new ideas, it is the grandmothers who are key decision makers around delivery and newborn care and their inclusion is vital.

## Conclusion

As home delivery is common and hospital stays are short, the expansion of outreach education and care represents an effective way of reaching more mothers and babies and improving newborn care. The considerable impact of a single ANM, very active in the community clearly demonstrates the progress that could be made with competent and dedicated outreach workers in this area. Research based on the organization SEARCH's model using village health workers [14] has shown the potential impact of community based workers more widely, and prompted development of IMNCI and the National Rural Health Mission. As in this study, the coverage of ANM and Anganwadi outreach has also been shown to be low in an area of rural Uttar Pradesh [34]. Improved coverage, timing and informational content of these workers' outreach is needed, with a particular focus on community education and behaviour change communication. Social-cognitive theory recognises that interaction with people rather than the provision of information alone, is the key to behaviour change especially if the latter conflicts with existing motives, beliefs and values [38]. The use of participatory health promotion techniques, such as women's groups, has proved very successful at reducing neonatal mortality elsewhere in South Asia, and assisting communities to find locally adapted solutions to problems [15].

Behaviour change messages need to be based on an understanding of the beliefs supporting traditional behaviour, and adapted and tailored to the specific environment. In understanding reactions to the essential newborn care package of interventions, the Innovation Diffusion theory is useful. It describes the process by which an innovation, new ideas, opinions, attitudes, and behaviours- are communicated through certain channels over time, and spread among the members of a social system or community. Its five major assumptions are that: adoption takes time; people pass through various stages in the adoption process; they can modify the innovation and sometimes discontinue its use; the perceived characteristics of the innovation influence its adoption; and individual characteristics influence its adoption [39]. In encouraging and directing behaviour change existing views need to be engaged with respectfully, health workers cannot see the community as an empty vessels into which to impart education. Messages can be adapted so that proposed changes relate to existing health worries, for example building on the local concern about babies catching cold. Where examples of positive deviance are present, these can be highlighted and affirmed. Also in some circumstances appropriate compromises between existing and recommended practices can be suggested for areas of care met with particular resistance.

Karnataka is now beginning to implement the NRHM but ASHAs were not in place at the time of this research. Their addition will create additional capacity but also challenges in ensuring effective collaborative working. It is vital that the ANMs, Anganwadi, and ASHAs who are collectively responsible for improving newborn care in rural India, are supported to develop effective, locally tailored behavior change communication strategies. To build on local understanding and beliefs that aim to protect the wellbeing of mother and child during the culturally important time of childbirth and become brokers of positive behavior change.

## Competing interests

The authors declare that they have no competing interests.

## Authors' contributions

AK carried out this work as part of the research for her PhD thesis. She led the analysis of the quantitative data and the collection and analysis of the qualitative data. She also drafted the manuscript. JC made an important contribution to the design of the study and the data analysis. He made substantial comments and revisions to the draft. AK and JC both read and approved the final manuscript.

## Pre-publication history

The pre-publication history for this paper can be accessed here:


